# Sitting less and moving more: the impact of physical activity on mortality in the population of Spain

**DOI:** 10.1186/s12889-024-20600-y

**Published:** 2024-11-12

**Authors:** Miguel Á de la Cámara, Cristina Ortiz, Beatriz Granero-Melcon, Alejandro Martínez-Portillo, Montserrat Neira-León, Iñaki Galán

**Affiliations:** 1 Department of Educational Sciences, University of Alcalá (UAH), Madrid, Spain; 2 National Centre for Epidemiology, Instituto de Salud Carlos III (ISCIII), Madrid, Spain; 3 National School of Health, Instituto de Salud Carlos III, Madrid, Spain; 4 Service of Epidemiology, General Directorate of Public Health and Addictions, Región de Murcia, Spain; 5 Ministry of Health, Madrid, Spain; 6 Department of Preventive Medicine and Public Health, Autonomous University of Madrid, Madrid, Spain; 7 National Centre for Epidemiology, Institute of Health Carlos III, Madrid, Spain

**Keywords:** Sedentarism, Movement, Lifestyles, Cohort study, Public health

## Abstract

**Background:**

Sitting time (ST) constitutes a significant aspect of sedentary behavior, and its worldwide escalation raises concerns regarding public health. International guidelines recommend limiting sedentary time and replacing it with physical activity (PA) to reduce the risk of diseases and mortality. This study examines the impact of replacing ST with PA on all-cause, cardiovascular disease (CVD), and cancer mortality in a representative cohort of the population of Spain.

**Methods:**

We included 30 955 participants aged 15–69 years from two National Health Surveys performed in 2011 and 2017. Data were linked to mortality records as of December 2022. Data on ST, light PA (LPA), and moderate-vigorous PA (MVPA) were collected as part of the International Physical Activity Questionnaire at baseline. Isotemporal substitution analysis from Poisson regression models was used to estimate the relative risk ratio (RR) of replacing ST with LPA or MVPA.

**Results:**

During a median follow-up of 5.7 years, 957 deaths were reported. The replacement of 1 h per week of ST with 1 h per week of MVPA was significantly associated with a lower risk of all-cause (3.3%), CVD (6.7%), and cancer mortality (3.1%). Similarly, replacing 1 h per week of ST with 1 h per week of LPA was significantly associated with a lower risk of all-cause (1.6%) and cancer mortality (2.1%). Finally, substituting 1 h per week of LPA with 1 h per week of MVPA was significantly associated with a 7.6% lower risk of CVD mortality.

**Conclusions:**

Substituting one hour per week of ST with an equivalent amount of PA was associated with a lower risk of all-cause, CVD, and cancer mortality.

## Background

Sedentary behavior (SB) is defined as any waking behavior characterized by an energy expenditure ≤ 1.5 metabolic equivalents (METs) while in a sitting, reclining, or lying posture [1]. Sitting time (ST) involves multiple occupational and non-occupational daily activities and is a major contributor to SB and, thus, it is commonly used as an SB measure in population-based studies [2, 3]. Worldwide, the trend towards SB is increasing [4], likely negatively impacting the world´s population health as time spent in sedentary activities results in detrimental and independent health effects regardless of physical activity levels [5]. Not surprisingly, PA and health international guidelines recommend limiting the amount of sedentary time [6–8] based on the wealth of scientific evidence showing associations between sedentarism and poor cardiometabolic health [9], certain types of cancer [10], and mortality [11].

However, merely reducing sedentary time might not improve mortality, as it may be necessary to replace SB with PA of any intensity [12]. In fact, based on strong evidence suggesting PA´s health benefits [13], PA guidelines recommend replacing sedentary time with additional PA of any intensity [6–8]. Unfortunately, the reality is the low prevalence of sufficient PA worldwide [14], which may significantly reduce the potential benefits of PA on health, as well as increase the risk of non-communicable diseases and mortality [15].

A growing body of research has investigated the replacement of sedentary time with PA using isotemporal substitution models (ISM), which estimate the effect of replacing one behavior (e.g., sitting time) with any PA for the same amount of time [16]. Grgic et al. [17] conducted a systematic review of studies using ISM, and their results suggested the substantial benefits of replacing time spent in SB with PA based on the favorable impact on health-related quality of life, mental health, adiposity, fitness, cardiometabolic biomarkers, chronic diseases, and other medical conditions [17]. In addition, it was observed that replacing SB with different PA levels (light or moderate-vigorous) was associated with a substantial reduction in the mortality risk ranging from 12 to 81% [12, 18–21]. This application of ISM allowed a quantitative approximation of the potential health effects of this behavioral change. These results could inform and make PA guidelines more effective, as well as improve and reinforce public health messages [17] aimed at reducing physical inactivity worldwide.

However, ISM studies with mortality as their outcome are burdened by several limitations and knowledge gaps. First, prospective population-based studies are scarce. Second, the theoretical behavior change most commonly proposed in the ISM studies—replacing behaviors for 30 min per day—may seem unfeasible for a large portion of the population. A proposed change that aligns more closely with PA global recommendations for adults, usually given as weekly recommendations [6–8], may feel more attainable and, thus, make for a more successful public health message. Finally, few of these studies compare the substitution effect of ISM among all-cause, CVD, and cancer mortality, as well as across several sociodemographic determinants.

Therefore, the purpose of this study was to estimate the theoretical impact of replacing one hour per week of SB (i.e., sitting time) with the equivalent of light PA (LPA) or moderate-vigorous PA (MVPA) on all-cause, CVD, and cancer mortality in a representative cohort of the Spanish population. Further, we examined such theoretical effects by sex, age, and educational level.

## Methods

### Study design and population

The National Health Interview Survey comprises a series of assessments which embody the main source of information on the health of the population of Spain. The survey collects population-based data on health status, behavioral, social, and environmental determinants of health, and use of health services. The participants are selected using a stratified multi-stage sampling and are representative of the non-institutionalized Spanish population [22]. The data for this study came from the 2011 and 2017 surveys, which included 21 007 and 23 089 participants over 15 years of age, respectively. Both surveys used the same sample design and standardized questionnaires administered face-to-face using a Computer-Assisted Personal Interview. Response rates were 66.5% and 69.9% of all selected households in 2011 and 2017 surveys, respectively.

This study was approved by Carlos III Institute of Health Ethical Research Committee (CEI PI 28_2019). All participants gave informed consent to participate in the study before being included.

### Variables

#### Mortality

Survey data were linked to mortality records through December 31, 2022, by the Spanish National Institute of Statistics using the national identification document number. Data on causes of death were collected according to ICD-10 [23]. Cancer mortality and cardiovascular disease mortality (CVD mortality) were defined by the C00-D48 and IC00-I99 codes, respectively.

#### Physical activity and sitting time

Physical activity and ST were assessed with the International Physical Activity Questionnaire Short-Form (IPAQ-SF). This 9-item questionnaire records the PA performed in the last 7 days corresponding to four intensity levels (sitting, walking, moderate-intensity, and vigorous-intensity activities) derived from four PA domains (leisure time PA, domestic activities, work-related PA, transport-related PA). The outcomes are the weekly time spent engaged in PA, sitting, MET min/week, and PA categories [24]. For this study, we used three categories from the IPAQ-SF: ST, LPA, and MVPA. To minimize bias from individuals who were inactive because they were sick or physically impaired, participants who reported very poor self-perceived health or severe functional limitations at baseline were excluded from the analyses.

#### Study covariates

The following demographic and healthy lifestyle variables were included as covariates: age (15–24, 25–34, 35–44, 35–44, 45–54, 55–64, 65–69 years old); sex (women and men); country of birth (Spain and other countries); educational level (elementary or lower, middle school, high school graduate, and university education); tobacco use (never, former, current < 15 cigs/day, and current ≥ 15 cigs/day); alcohol intake based on both frequency of consumption and volume consumed (never drinkers, former drinkers, occasional drinkers, drinkers > 0–20 g/day, and drinkers > 20 g/day); binge drinking (yes/no) defined as the consumption of ≥ 6 or ≥ 5 standard drinks (10 g of ethanol) within a 4–6 h period for men and women, respectively; self-reported body mass index (BMI underweight < 18.5 kg/m^2^, normal weight 18.5–24.9 kg/m^2^, overweight 25.0–29.9 kg/m^2^, obesity $\:\ge\:$30 kg/m^2^, and not available); and level of adherence to a high quality diet based on the Mediterranean Diet Adherence Screener index, ranging from 0 (lowest adherence) to 10 (highest adherence). Participants were classified as having either low, medium, or high adherence [25, 26].

### Statistical analysis

Data analyses were conducted on 30 955 individuals from the total sample of 44 096 participants, selected based on age restrictions outlined in the IPAQ-SF (between 15 and 69 years old) and the availability of valid data.

The study involved analyzing the distribution of categorical data and computing proportions along with their corresponding 95% confidence intervals (CI) across categories within the dataset. Additionally, continuous PA variables were represented by weighted means and standard deviations (SD). To ascertain differences between descriptive variables, statistical analyses including t-tests and analysis of variance (ANOVA) were employed.

Three different Poisson regression models were fitted to estimate the incidence rate ratios (IRR) and 95% CI of the association between each PA/ST and the risk of death (all-cause mortality, cancer mortality, and CVD mortality). First, the single model estimated the association between mortality and each PA or ST separately, and is expressed as follows:

Mortality (IRR) = (b_1_) LPA + (b_5_) covariates.

Second, the partition model represented the effect of increasing the activity type while holding other activity types constant, and is expressed as follows:

Mortality (IRR) = (b_1_) LPA + (b_2_) MVPA + (b_3_) ST + (b_5_) covariates.

Third, the hypothetical effect of replacing ST with PA (LPA or MVPA) on mortality was estimated with the ISM. Likewise, the substitution of LPA with MVPA maintaining the ST was also evaluated. This method estimates the effects of replacing one activity for another for the same amount of time (1 h/week, in this case), and is expressed as follows:

Mortality (IRR) = (b_1_) LPA + (b_2_) MVPA + (b_4_) total time + (b_5_) covariates.

where the coefficients b_1_ and b_2_ represent the effects of 1 h/week substitution of ST with LPA or MVPA while the total time remains constant. The analyses were conducted for the whole sample and then stratified by sex. All models were adjusted for sex, age, country of birth, educational level, tobacco use, alcohol intake, binge drinking, BMI, adherence to Mediterranean diet.

Analyses were performed with the STATA version 18.0 (StataCorp LLC, College Station, TX, USA), and the survey command was used to consider the complex design of the survey.

## Results

Descriptive characteristics of the sample and time spent on PA levels and ST are shown in Table 1. During a median follow-up of 5.7 years, 957 participants died (310 women and 647 men). The leading causes of death were cancer (460 participants), and CVD (204 individuals). Participants reported an average ST of 33 h/week, 6.2 h/week of LPA, and 2.8 h/week of MVPA.

**Table 1:** Descriptive characteristics of the sample according to physical activity levels and sitting time

	Total		Light PA	Moderate- vigorous PA	Sitting time
Variable	*N* ^a^	Proportion %^b^	Mean (SD) ^b^ hours/week	Mean (SD) ^b^ hours/week	Mean (SD) ^b^ hours/week
Total	30,955		6.2(6.3)	2.8(5.3)	33.0(18.9)
Sex					
Women	16,002	50.0	6.0(6.1)†	2.1(4.4)‡	31.7(18.3)‡
Men	14,953	50.0	6.3(6.6)	3.6(5.9)	34.3(19.4)
Age					
15–24	3030	13.8	5.9(6.3)‡	4.0(5.7)‡	42.2(20.1)‡
25–34	4563	18.7	6.2(6.6)	3.2(5.3)	33.3(20.1)
35–44	7433	23.6	5.8(6.4)	2.9(5.2)	32.0(19.6)
45–54	6916	21.1	6.1(6.2)	2.6(5.2)	32.0(18.8)
55–64	6209	16.1	6.5(6.3)	2.4(5.2)	31.1(16.7)
65–69	2804	6.6	6.8(6.1)	2.2(4.9)	31.1(14.8)
Country of birth					
Spain	27,496	84.1	6.1(6.3)†	2.8(5.2)	33.5(19.0)‡
Other	3459	15.9	6.5(6.6)	2.8(5.5)	28.8(17.0)
Level of education					
Elementary or lower	5207	15.4	6.1(6.4)‡	2.3(5.4)‡	29.5(16.2)‡
Secondary Education	9640	32.2	6.6(6.7)	3.0(5.8)	30.1(17.7)
High school	9877	32.7	6.2(6.4)	3.0(5.2)	33.2(19.2)
University	6231	19.7	5.5(5.7)	2.7(4.3)	39.9(20.2)
Smoking					
Never smokers	14,789	49.4	6.0(6.2)†	2.9(5.2)‡	33.3(18.9)‡
Former smokers	7052	21.5	6.4(6.3)	2.9(5.3)	33.6(19.1)
Smokers 1–14 cig/day	5681	18.5	6.4(6.5)	2.7(5.1)	31.8(18.4)
Smokers ≥ 15 cig/day	3433	10.7	6.0(6.7)	2.5(5.8)	32.1(18.9)
Alcohol intake					
Never drinkers	5508	18.9	6.4(6.4)‡	2.6(5.4)‡	31.1(18.1)‡
Formers drinkers	3596	10.9	5.9(6.1)	2.3(5.0)	31.4(18.2)
Occasional drinkers	9402	31.0	6.0(6.3)	2.7(5.0)	33.3(19.3)
Drinkers > 0–20 g/day	10,503	33.4	6.3(6.3)	3.0(5.3)	34.1(19.0)
Drinkers > 20 g/day	1946	5.9	6.5(6.7)	3.7(6.5)	33.3(18.8)
Binge drinking					
No	28,757	92.7	6.2(6.3)†	2.8(5.2)‡	32.7(18.7)‡
Yes	2198	7.3	5.8(6.4)	3.5(5.8)	36.5(20.2)
Body mass index					
Normal weight	13,705	46.0	6.2(6.3)†	3.1(5.1)‡	33.3(19.2)‡
Overweight	10,782	33.5	6.3(6.4)	2.9(5.5)	32.2(18.4)
Underweight	670	2.5	5.9(6.2)	2.5(4.8)	36.9(20.2)
Obesity	4725	14.7	5.9(6.3)	2.1(5.1)	33.4(18.6)
N/A	1073	3.2	5.2(5.9)	2.1(5.1)	31.0(18.3)
Adherence to Mediterranean Diet					
Very high	6445	18.9	7.3(6.5)‡	3.1(5.3)‡	31.3(17.9)‡
High	5022	15.4	6.3(6.2)	2.7(5.0)	32.3(18.5)
Low	5780	18.3	6.2(6.3)	2.7(5.1)	32.8(18.6)
Very low	13,708	47.4	5.6(6.3)	2.8(5.4)	34.0(19.4)
All-cause mortality					
No	29,998	97.5	6.2(6.3)	2.9(5.3)‡	32.9(18.9)†
Yes	957	2.5	6.2(6.5)	1.8(4.8)	34.8(18.8)
CVD mortality					
No	30,751	99.5	6.2(6.3)	2.8(5.3)†	33.0(18.8)†
Yes	204	0.5	6.8(7.1)	1.3(4.1)	36.7(20.3)
Cancer mortality					
No	30,495	98.8	6.2(6.3)	2.9(5.3)‡	32.9(18.9)
Yes	460	1.2	6.0(6.1)	1.8(4.7)	34.0(18.7)

PA: Physical activity; SD: Standard deviation; CVD: Cardiovascular diseases; N/A: Data not available

ª Unweighted; ^b^ %/Weighted means

† P value < 0.05; ‡ P value < 0.001

Table 2 presents the single variable, partition, and isotemporal models examining the associations between PA levels and ST with all-cause, CVD, and cancer mortality. The adjusted partition model, where the models were mutually adjusted for all activity categories, showed that ST was associated with a higher risk for all-cause, CVD, and cancer mortality (1.0%, 1.4%, and 0.7% higher risk, respectively). In contrast, MVPA was associated with a lower risk for all-cause, CVD, and cancer mortality (2.4%, 4.5%, and 2.4% lower risk, respectively). Finally, LPA was not associated to any change in mortality risk.

**Table 2:** Associations among all-cause, cardiovascular disease, and cancer mortality, and physical activity levels and sitting time

	All-cause mortality	CVD mortality	Cancer mortality
	IRR(95%CI)	IRR(95%CI)	IRR(95%CI)
N deaths/total	957/30,955	204/30,955	460/30,955
Single model unadjusted			
MVPA	0.945 (0.922,0.969)	0.900 (0.833,0.971)	0.947 (0.915,0.980)
LPA	0.995 (0.983,1.007)	1.015 (0.989,1.042)	0.992 (0.976,1.007)
ST	1.006 (1.002,1.010)	1.009 (1.001,1.018)	1.004 (0.998,1.010)
Single model adjusted^a^			
MVPA	0.970 (0.953,0.989)	0.941 (0.893,0.993)	0.970 (0.945,0.996)
LPA	0.986 (0.974,0.999)	1.011 (0.983,1.040)	0.979 (0.963,0.996)
ST	1.011 (1.006,1.015)	1.013 (1.004,1.022)	1.009 (1.002,1.016)
Partition model unadjusted			
MVPA	0.946 (0.923,0.970)	0.899 (0.832,0.971)	0.948 (0.916,0.981)
LPA	1.002 (0.991,1.014)	1.027 (1.001,1.054)	0.997 (0.981,1.013)
ST	1.005 (1.001,1.009)	1.010 (1.001,1.019)	1.003 (0.997,1.009)
Partition model adjusted^a^			
MVPA	0.976 (0.958,0.995)	0.945 (0.897,0.997)	0.976 (0.950,1.002)
LPA	0.994 (0.981,1.007)	1.024 (0.995,1.053)	0.986 (0.968,1.003)
ST	1.010 (1.005,1.014)	1.014 (1.005,1.023)	1.007 (1.000,1.014)
Isotemporal unadjusted			
MVPA x ST	0.941 (0.918,0.966)	0.890 (0.823,0.962)	0.946 (0.913,0.979)
LPA x ST	0.997 (0.985,1.009)	1.017 (0.991,1.044)	0.994 (0.979,1.010)
MVPA x LPA	0.944 (0.919,0.971)	0.875 (0.807,0.948)	0.951 (0.915,0.988)
Isotemporal adjusted^a^			
MVPA x ST	0.967 (0.949,0.985)	0.933 (0.885,0.983)	0.969 (0.944,0.995)
LPA x ST	0.984 (0.972,0.997)	1.010 (0.982,1.038)	0.979 (0.962,0.996)
MVPA x LPA	0.982 (0.961,1.004)	0.924 (0.869,0.981)	0.990 (0.959,1.023)

Incidence rate ratio (IRR) and 95% confidence interval (95%CI)

CVD: Cardiovascular diseases; MVPA: moderate-vigorous physical activity; LPA: light physical activity; ST: sitting time

Single model examining the association of each activity individually with all-cause, CVD, and cancer mortality

Partition model examining the association of a 1 h/week increase in each activity while holding other activity types constant with all-cause, CVD, and cancer mortality

Isotemporal model examining the association of replacing 1 h/week of one activity type with 1 h/week of another activity type with all-cause, CVD, and cancer mortality

^a^ Adjusted for sex, age, country of birth, educational level, smoking, alcohol intake, binge drinking, body mass index, and adherence to Mediterranean Diet

Based on the isotemporal analysis (adjusted models), substituting ST with PA reduced mortality risk. The replacement of 1 h/week of ST with 1 h/week of MVPA was significantly associated with lower risk for all-cause, CVD, and cancer mortality (3.3%, 6.7%, and 3.1%, respectively). Likewise, replacing 1 h/week of ST with 1 h/week of LPA was significantly associated with lower risk for all-cause and cancer mortality (1.6 and 2.1%, respectively) with no effect on CVD mortality. In turn, increasing PA intensity did impact CVD mortality. Substituting 1 h/week of LPA with 1 h/week of MVPA was significantly associated with a 7.6% lower risk of CVD mortality.

In Table 3 we reran these analyses stratified by sociodemographic variables. Although replacing 1 h/week of ST with the same amount of time of MVPA had a protective effect independent of sex, age, and educational level, the size of the effect varies. The favorable impact is stronger in women than in men regarding all-cause (8.5% vs. 2.0%) and cancer mortality (11.1% vs. 1.3%). It is also stronger for CVD mortality among individuals with higher versus lower educational level (12.1% vs. 1.4%).

**Table 3:** Isotemporal substitution of sitting time with physical activity levels, and risk for all-cause, cardiovascular diseases, and cancer mortality, by sex, age, and educational level

		ST with MVPA		ST with LPA		LPA wit MVPA	
	*N* deaths/total	IRR (95%CI)	*P* for interaction	IRR (95%CI)	*P* for interaction	IRR (95%CI)	*P* for interaction
**All-cause mortality**							
Sex							
Women	310/16,002	0.915 (0.871,0.962)		0.975 (0.949,1.001)		0.939 (0.885,0.996)	
Men	647/14,953	0.980 (0.961,0.999)	0.019	0.989 (0.975,1.004)	0.387	0.990 (0.967,1.014)	0.114
Age							
15–54 years	252/21,942	0.972 (0.939,1.006)		0.999 (0.975,1.023)		0.973 (0.934,1.014)	
55–69 years	705/9013	0.963 (0.942,0.985)	0.765	0.977 (0.963,0.991)	0.155	0.986 (0.960,1.013)	0.604
Educational level							
Secondary or higher education	621/25,748	0.964 (0.941,0.987)		0.978 (0.963,0.994)		0.985 (0.957,1.013)	
Primary education or no education	336/5207	0.972 (0.943,1.002)	0.729	0.999 (0.978,1.020)	0.179	0.973 (0.937,1.011)	0.660
**CVD mortality**							
Sex							
Women	67/16,002	0.853 (0.759,0.958)		1.027 (0.978,1.079)		0.830 (0.726,0.948)	
Men	137/14,953	0.945 (0.893,1.000)	0.108	1.004 (0.971,1.038)	0.579	0.941 (0.881,1.006)	0.101
Age							
15–54 years	49/21,942	0.889 (0.808,0.978)		1.054 (1.001,1.109)		0.843 (0.756,0.941)	
55–69 years	155/9013	0.946 (0.892,1.004)	0.237	0.988 (0.957,1.020)	0.051	0.958 (0.897,1.023)	0.059
Educational level							
Secondary or higher education	128/25,748	0.879 (0.815,0.947)		1.002 (0.967,1.039)		0.877 (0.804,0.957)	
Primary education or no education	76/5207	0.986 (0.929,1.047)	0.036	1.027 (0.980,1.078)	0.612	0.960 (0.885,1.041)	0.133
**Cancer mortality**							
Sex							
Women	163/16,002	0.889 (0.818,0.968)		0.950 (0.917,0.984)		0.936 (0.850,1.032)	
Men	297/14,953	0.987 (0.961,1.014)	0.031	0.992 (0.972,1.012)	0.048	0.995 (0.962,1.030)	0.316
Age							
15–54 years	107/21,942	0.946 (0.882,1.015)		0.983 (0.952,1.016)		0.962 (0.889,1.042)	
55–69 years	353/9013	0.977 (0.950,1.006)	0.353	0.977 (0.957,0.998)	0.929	1.000 (0.963,1.038)	0.406
Educational level							
Secondary or higher education	310/25,748	0.962 (0.931,0.995)		0.970 (0.949,0.991)		0.992 (0.954,1.032)	
Primary education or no education	150/5207	0.981 (0.937,1.028)	0.561	0.999 (0.969,1.029)	0.138	0.983 (0.925,1.044)	0.766

Isotemporal substitution examining the association of replacing 1 h/week of one activity type with 1 h/week of another activity type

MVPA: moderate-vigorous physical activity; LPA: light physical activity; ST: sitting time; CVD: Cardiovascular disease

Incidence rate ratio (IRR) and 95% confidence interval (95%CI). P value for interactions with sex, age, and educational level categories

All models were adjusted for sex, age, country of birth, educational level, smoking, alcohol intake, binge drinking, body mass index, and adherence to Mediterranean Diet

Similarly, the substitution of 1 h/week of ST with 1 h/week of LPA had a stronger protective effect in women than in men but only for cancer mortality (5% vs. 0.8%). Finally, the impact of replacing LPA with MVPA did not vary by sex, age, and educational level in its associations with all-cause, CVD, and cancer mortality (p for interaction > 0.05).

## Discussion

Our study provides novel insights into the potential mortality benefits derived from substituting ST with different levels of PA in a population-based cohort of adults residing in Spain. Our results suggest that replacing 1 h/week of ST with an equivalent time of PA reduces the risk for all-cause, CVD, and cancer mortality. The encouraging association was observed when replacing ST with either LPA or MVPA, and also when increasing PA intensity from LPA to MVPA. Thus, although our findings strongly suggest that any increase in PA may benefit health, they also show that the strengths of such associations do vary by type of mortality risk, i.e., all-cause, CVD, or cancer mortality. Further, our stratified results indicate that the magnitude of some of these relationships may vary by sex, age, and educational level.

Our findings support previous research on the beneficial impact on mortality risk stemming from replacing sedentary time with PA [12, 18–21, 27–29]. Nonetheless, we observed a smaller reduction in mortality risk compared to these previous studies, which reported a reduction ranging from 12 to 81%. This discrepancy may be attributed to our use of different methods for estimating SB and PA, as most previous studies utilized objective methods such as accelerometers [12, 18–21, 27, 28].

Nevertheless, their results should be interpreted cautiously, as they involved short follow-up periods, older adults, or limited statistical adjustments for poor health. These factors could result in an overestimation of the association strength between PA and mortality, especially with MVPA [30]. In addition, it has been suggested that self-reported methods may underestimate the strength of these relationships and the true reduction in mortality risk [31, 32]. Therefore, one may argue that had we used objective methods in this study, the associations observed here would have been amplified and thus closer to those referred by these studies.

Furthermore, it is worth noting that our study assessed only ST, whereas other works have included various SB measured by accelerometers or other PA questionnaires. These methods may capture different types of SB related to mortality, such as lying down [33]. Alternatively, the lower impact on mortality observed in our study may be related to the different substitution time proposed. Most ISM studies proposed 30 min/day [12, 19–21, 27, 28], whereas our study proposed 1 h/week. This shorter substitution time could have attenuated the magnitude of these associations, indicating a dose-response association between the PA volume and health outcomes, as reported previously [34, 35].

Our results also suggest that the replacement of 1 h/week of ST with 1 h/week of MVPA versus LPA provides greater mortality risk reduction. This is consistent with previous research reporting a stronger association with mortality when substituting SB with MVPA rather than with LPA [12, 18, 19, 27, 28, 36].

However, we found that the substitution of ST with LPA was not associated to CVD mortality. Although LPA has been associated with improved cardiometabolic health and reduced mortality risk [37], our results indicated that LPA might not be sufficient to counteract the effects of SB on CVD mortality as it falls short of providing the significant cardiovascular benefits that come with MVPA. Interestingly, our findings also show that replacing LPA with MVPA was associated with a risk reduction in only CVD mortality. A plausible explanation would be that MVPA activates a range of higher or more CVD-relevant physiological responses than LPA. These responses would, in turn, cause a more significant metabolic favorable impact on cardiovascular health thus counteracting the negative metabolic effects of SB [38]. Thus, these findings highlight the potential beneficial impact of replacing sitting time or LPA with MVPA to lower the risk of CVD-related deaths.

Nevertheless, our results indicate that replacing SB with LPA could also reduce the risk of mortality. Specifically, this substitution was associated with a lower risk for all-cause and cancer mortality. In fact, we observed that substituting ST with LPA might confer protective effects against cancer-related mortality, with the reduction being only slightly under 1% point than that achieved by substituting ST with MVPA. Generally, the associations between PA and cancer mortality are weaker and more heterogeneous compared to other causes of mortality. The heterogeneity might result from grouping different cancer types together. Also, different PA levels could affect individuals differently depending on the cancer type or site [39, 40].

Our results underscore the need for further investigation and are highly relevant for improving public health messaging. This work suggest that small changes in PA and SB can protect against mortality related to a range of chronic diseases, including cancer mortality [6, 35]. This reinforces the World Health Organization’s message, based on the current scientific paradigm, that every move counts towards better health [41], and may motivate individuals unable to perform MVPA to engage in LPA. These individuals may find it more feasible to replace SB with LPA, rather than with MVPA, thus improving their health.

Our analyses stratified by sociodemographic variables show that higher educated individuals could derive more benefits from substituting ST with MVPA than their lower educated counterparts. We would argue that those with higher educational level often hold higher-ranking positions involving prolonged ST and thus at a higher mortality risk [42]. Thereby, substituting sedentary time with PA might help attenuate this risk in this subpopulation [43].

Further, health-related benefits of PA differ by PA domains, with greater benefits associated with PA performed during leisure time (LTPA) [44, 45]. Considering the educational disparities in reported PA domains— where higher educated individuals are more likely to engage in LTPA [46, 47] than others—could help explain the observed differences in benefits.

Reported sex differences in response to PA, reveal that women experience more significant reductions than men in overall and cardiovascular mortality risk at equivalent levels of LTPA [48, 49]. To the best of our knowledge, only two studies have analyzed the substituting effects of replacing SB with PA on mortality risk by sex. One study [27] found similar results across sexes when replacing 30 min/day of ST with LPA or MVPA for all-cause, CVD, and cancer mortality. However, another study [28] showed that replacing 30 min/day of SB with an equal amount of time of MVPA was associated with a lower risk for all-cause mortality in men, but not in women. Our results indicated that the effects of replacing 1 h/week of SB with 1 h/week of PA (LPA or MVPA) might confer more protection against all-cause, CVD, and cancer mortality in women than in men. Overall, this indicates the importance of considering and further exploring sex interaction effects in these associations in future studies.

This study has several strengths. We used data from two nationally representative samples of the population residing in Spain over 15 years of age, and followed-up for a median of 5.7 years. To the best of our knowledge, this is the first study to estimate the substitution effects of replacing ST time with an equal amount of PA time in the population of a Southern European country. In addition, we controlled for a wide range of potential confounding variables and excluded participants reporting very poor self-perceived health or severe functional limitations at baseline. Furthermore, unlike previous studies, our proposed short substitution time of ST for PA (1 h/week) to achieve mortality benefits makes our findings easily transferable to PA guidelines [6–8] and may inform a more efficacious public health message.

However, our study was not without limitations. Both PA and ST were self-reported measures, susceptible to recall bias, which may have underestimated SB and overestimated PA [50, 51]. Although the IPAQ-SF might overestimate PA [52], it is recommended as a cost-effective method for large-scale monitoring of PA in the EU [53] given its acceptable measurement properties [24]. In contrast, the single-item SB question from the IPAQ-SF had low criterion validity and provided limited information [54]. Having said that, single-item measures can be a valid screening tool to determine whether respondents´ activity levels are sufficient to provide health benefits [55]. Moreover, objective methods are not without limitations [56], they present their own challenges, and they are difficult to implement in the context of large-scale epidemiological studies [57]. Further, PA and ST were reported only at baseline, and were not re-assessed, so follow-up data on these behaviors were not available. In addition, these findings are based on theoretical models. However, ISM has been suggested as a useful method to discern the morbidity and mortality benefits that could be achieved when SB is replaced with PA [58]. Finally, while our study was conducted in a representative Spanish population, the generalizability of the findings to other countries or cultural contexts may not be directly applicable. However, these findings may contribute to the public health message regarding the amount of time spent in SB that needs to be replaced with PA to confer health benefits.

## Conclusions

Our findings provide supporting evidence to support the message that less sitting time and more physical activity diminishes mortality risk. Our results indicate that replacing 1 h per week of sitting time with the same time of light physical activity or moderate-to-vigorous physical activity is significantly associated with a reduction in all-cause, CVD, and cancer mortality risk. Additionally, our results indicate that the magnitude of these effects may vary according to certain sociodemographic variables, especially by sex. Future research should focus on the mechanisms by which the substitution of ST with different PA levels reduces the risk of mortality for various causes of death, as well as the source of the sex difference.

## Data Availability

The data supporting the findings of this study are available from the Spanish Ministry of Health and the Spanish National Institute of Statistics. Access to these data is restricted, as they were used under specific collaboration agreements and conventions for this study and are therefore not publicly available.

## Declarations

### Disclaimer

This article presents independent research. The views expressed are those of the authors and not necessarily those of the Institute of Health Carlos III.

## Abbreviations

ST: Sitting time
PA: Physical activity
CVD: Cardiovascular diseases
LPA: Light physical activity
MVPA: Moderate-vigorous physical activity
RR: Risk ratio
IRR: Incidence risk ratio
METs: Metabolic equivalent task
ISM: Isotemporal substitution models
IPAQ-SF: International Physical Activity Questionnaire Short-Form
BMI: Body mass index

## References

[1] Tremblay MS, Aubert S, Barnes JD, Saunders TJ, Carson V, Latimer-Cheung AE, et al. Sedentary Behavior Research Network (SBRN) – terminology Consensus Project process and outcome. Int J Behav Nutr Phys Act. 2017;14(1):75. 28599680 10.1186/s12966-017-0525-8 PMC5466781

[2] Healy GN, Clark BK, Winkler EAH, Gardiner PA, Brown WJ, Matthews CE. Measurement of adults’ sedentary time in Population-Based studies. Am J Prev Med. 2011;41(2):216–27. 21767730 10.1016/j.amepre.2011.05.005 PMC3179387

[3] Dunstan DW, Dogra S, Carter SE, Owen N. Sit less and move more for cardiovascular health: emerging insights and opportunities. Nat Rev Cardiol. 2021;18(9):637–48. 34017139 10.1038/s41569-021-00547-y

[4] Yang L, Cao C, Kantor ED, Nguyen LH, Zheng X, Park Y, et al. Trends in Sedentary Behavior among the US Population, 2001–2016. JAMA. 2019;321(16):1587–97. 31012934 10.1001/jama.2019.3636 PMC6487546

[5] Biswas A, Oh PI, Faulkner GE, Bajaj RR, Silver MA, Mitchell MS, et al. Sedentary time and its association with risk for disease incidence, mortality, and hospitalization in adults: a systematic review and meta-analysis. Ann Intern Med. 2015;162(2):123–32. 25599350 10.7326/M14-1651

[6] Bull FC, Al-Ansari SS, Biddle S, Borodulin K, Buman MP, Cardon G, et al. World Health Organization 2020 guidelines on physical activity and sedentary behaviour. Br J Sports Med. 2020;54(24):1451–62. 33239350 10.1136/bjsports-2020-102955 PMC7719906

[7] Piercy KL, Troiano RP, Ballard RM, Carlson SA, Fulton JE, Galuska DA, et al. The physical activity guidelines for americans. JAMA. 2018;320(19):2020–8. 30418471 10.1001/jama.2018.14854 PMC9582631

[8] Ross R, Chaput JP, Giangregorio LM, Janssen I, Saunders TJ, Kho ME, et al. Canadian 24-Hour Movement guidelines for adults aged 18–64 years and adults aged 65 years or older: an integration of physical activity, sedentary behaviour, and sleep. Appl Physiol Nutr Metab Physiol Appl Nutr Metab. 2020;45(10):S57–102. 10.1139/apnm-2020-0467 33054332

[9] Bailey DP, Hewson DJ, Champion RB, Sayegh SM. Sitting time and risk of Cardiovascular Disease and Diabetes: a systematic review and Meta-analysis. Am J Prev Med. 2019;57(3):408–16. 31377090 10.1016/j.amepre.2019.04.015

[10] Hermelink R, Leitzmann MF, Markozannes G, Tsilidis K, Pukrop T, Berger F, et al. Sedentary behavior and cancer-an umbrella review and meta-analysis. Eur J Epidemiol. 2022;37(5):447–60. 35612669 10.1007/s10654-022-00873-6 PMC9209390

[11] Chau JY, Grunseit AC, Chey T, Stamatakis E, Brown WJ, Matthews CE, et al. Daily sitting time and all-cause mortality: a meta-analysis. PLoS ONE. 2013;8(11):e80000. 24236168 10.1371/journal.pone.0080000 PMC3827429

[12] Diaz KM, Duran AT, Colabianchi N, Judd SE, Howard VJ, Hooker SP. Potential effects on Mortality of replacing sedentary time with short sedentary bouts or physical activity: a National Cohort Study. Am J Epidemiol. 2019;188(3):537–44. 30551177 10.1093/aje/kwy271 PMC6395167

[13] Posadzki P, Pieper D, Bajpai R, Makaruk H, Könsgen N, Neuhaus AL, et al. Exercise/physical activity and health outcomes: an overview of Cochrane systematic reviews. BMC Public Health. 2020;20(1):1724. 33198717 10.1186/s12889-020-09855-3 PMC7670795

[14] Guthold R, Stevens GA, Riley LM, Bull FC. Worldwide trends in insufficient physical activity from 2001 to 2016: a pooled analysis of 358 population-based surveys with 1·9 million participants. Lancet Glob Health. 2018;6(10):e1077–86. 30193830 10.1016/S2214-109X(18)30357-7

[15] Lee IM, Shiroma EJ, Lobelo F, Puska P, Blair SN, Katzmarzyk PT. Effect of physical inactivity on major non-communicable diseases worldwide: an analysis of burden of disease and life expectancy. Lancet Lond Engl. 2012;380(9838):219–29. 10.1016/S0140-6736(12)61031-9 PMC3645500 22818936

[16] Mekary RA, Willett WC, Hu FB, Ding EL. Isotemporal substitution paradigm for physical activity epidemiology and weight change. Am J Epidemiol. 2009;170(4):519–27. 19584129 10.1093/aje/kwp163 PMC2733862

[17] Grgic J, Dumuid D, Bengoechea EG, Shrestha N, Bauman A, Olds T, et al. Health outcomes associated with reallocations of time between sleep, sedentary behaviour, and physical activity: a systematic scoping review of isotemporal substitution studies. Int J Behav Nutr Phys Act. 2018;15(1):69. 30001713 10.1186/s12966-018-0691-3 PMC6043964

[18] Fishman EI, Steeves JA, Zipunnikov V, Koster A, Berrigan D, Harris TA, et al. Association between objectively measured physical activity and mortality in NHANES. Med Sci Sports Exerc. 2016;48(7):1303–11. 26848889 10.1249/MSS.0000000000000885 PMC4911242

[19] Loprinzi PD, Loenneke JP. Mortality risk and perceived quality of life as a function of waking time in discretionary movement-based behaviors: isotemporal substitution effects. Qual Life Res Int J Qual Life Asp Treat Care Rehabil. 2017;26(2):343–8. 10.1007/s11136-016-1385-4 27495275

[20] Dohrn IM, Kwak L, Oja P, Sjöström M, Hagströmer M. Replacing sedentary time with physical activity: a 15-year follow-up of mortality in a national cohort. Clin Epidemiol. 2018;10:179–86. 29416378 10.2147/CLEP.S151613 PMC5790069

[21] Sun Y, Chen C, Yu Y, Zhang H, Tan X, Zhang J, et al. Replacement of leisure-time sedentary behavior with various physical activities and the risk of dementia incidence and mortality: a prospective cohort study. J Sport Health Sci. 2023;12(3):287–94. 36379419 10.1016/j.jshs.2022.11.005 PMC10199132

[22] de Sanidad M, Servicios Sociales e Igualdad. National Health Survey. 2011. Methodology [Internet]. 2011 [cited 2023 Aug 18]. https://www.sanidad.gob.es/en/estadEstudios/estadisticas/encuestaNacional/encuestaNac2011/MetodologiaENSE2011_12.pdf

[23] Instituto Nacional de Estadística. Death statistics according to cause of death. Methodology. [Internet]. 2023 [cited 2023 Aug 18]. https://www.ine.es/dyngs/INEbase/es/operacion.htm?c=Estadistica_C&cid=1254736176780&menu=metodologia&idp=1254735573175

[24] Craig CL, Marshall AL, Sjöström M, Bauman AE, Booth ML, Ainsworth BE, et al. International physical activity questionnaire: 12-country reliability and validity. Med Sci Sports Exerc. 2003;35(8):1381–95. 12900694 10.1249/01.MSS.0000078924.61453.FB

[25] Schröder H, Fitó M, Estruch R, Martínez-González MA, Corella D, Salas-Salvadó J, et al. A short screener is valid for assessing Mediterranean diet adherence among older Spanish men and women. J Nutr. 2011;141(6):1140–5. 21508208 10.3945/jn.110.135566

[26] Ortiz C, López-Cuadrado T, Rodríguez-Blázquez C, Pastor-Barriuso R, Galán I. Clustering of unhealthy lifestyle behaviors, self-rated health and disability. Prev Med. 2022;155:106911. 34922996 10.1016/j.ypmed.2021.106911

[27] Rees-Punia E, Evans EM, Schmidt MD, Gay JL, Matthews CE, Gapstur SM, et al. Mortality risk reductions for replacing Sedentary Time with Physical activities. Am J Prev Med. 2019;56(5):736–41. 30905483 10.1016/j.amepre.2018.12.006 PMC8485344

[28] Schmid D, Ricci C, Baumeister SE, Leitzmann MF. Replacing sedentary time with physical activity in relation to Mortality. Med Sci Sports Exerc. 2016;48(7):1312–9. 26918559 10.1249/MSS.0000000000000913

[29] Ekelund U, Steene-Johannessen J, Brown WJ, Fagerland MW, Owen N, Powell KE, et al. Does physical activity attenuate, or even eliminate, the detrimental association of sitting time with mortality? A harmonised meta-analysis of data from more than 1 million men and women. Lancet. 2016;388(10051):1302–10. 27475271 10.1016/S0140-6736(16)30370-1

[30] Matthews CE, Troiano RP, Salerno EA, Berrigan D, Patel SB, Shiroma EJ, et al. Exploration of confounding due to Poor Health in an accelerometer-mortality study. Med Sci Sports Exerc. 2020;52(12):2546–53. 32472927 10.1249/MSS.0000000000002405 PMC7669589

[31] Ramakrishnan R, He JR, Ponsonby AL, Woodward M, Rahimi K, Blair SN, et al. Objectively measured physical activity and all cause mortality: a systematic review and meta-analysis. Prev Med. 2021;143:106356. 33301824 10.1016/j.ypmed.2020.106356

[32] Celis-Morales CA, Perez-Bravo F, Ibañez L, Salas C, Bailey MES, Gill JMR. Objective vs. self-reported physical activity and sedentary time: effects of Measurement Method on relationships with Risk biomarkers. PLoS ONE. 2012;7(5):e36345. 22590532 10.1371/journal.pone.0036345 PMC3348936

[33] Holtermann A, Mork PJ, Nilsen TIL. Hours lying down per day and mortality from all-causes and cardiovascular disease: the HUNT Study, Norway. Eur J Epidemiol. 2014;29(8):559–65. 25027589 10.1007/s10654-014-9939-7

[34] Ekelund U, Tarp J, Steene-Johannessen J, Hansen BH, Jefferis B, Fagerland MW, et al. Dose-response associations between accelerometry measured physical activity and sedentary time and all cause mortality: systematic review and harmonised meta-analysis. BMJ. 2019;366:l4570. 31434697 10.1136/bmj.l4570 PMC6699591

[35] Garcia L, Pearce M, Abbas A, Mok A, Strain T, Ali S, et al. Non-occupational physical activity and risk of cardiovascular disease, cancer and mortality outcomes: a dose–response meta-analysis of large prospective studies. Br J Sports Med. 2023;57(15):979. 36854652 10.1136/bjsports-2022-105669 PMC10423495

[36] Matthews CE, Keadle SK, Troiano RP, Kahle L, Koster A, Brychta R, et al. Accelerometer-measured dose-response for physical activity, sedentary time, and mortality in US adults. Am J Clin Nutr. 2016;104(5):1424–32. 27707702 10.3945/ajcn.116.135129 PMC5081718

[37] Chastin SFM, De Craemer M, De Cocker K, Powell L, Van Cauwenberg J, Dall P, et al. How does light-intensity physical activity associate with adult cardiometabolic health and mortality? Systematic review with meta-analysis of experimental and observational studies. Br J Sports Med. 2019;53(6):370–6. 29695511 10.1136/bjsports-2017-097563 PMC6579499

[38] Nyssa T, Hadgraft E, Winkler RE, Climie, Megan S, Grace L, Romero N, Owen, et al. Effects of sedentary behaviour interventions on biomarkers of cardiometabolic risk in adults: systematic review with meta-analyses. Br J Sports Med. 2021;55(3):144. 32269058 10.1136/bjsports-2019-101154 PMC7841485

[39] Moore SC, Lee IM, Weiderpass E, Campbell PT, Sampson JN, Kitahara CM, et al. Association of leisure-time physical activity with risk of 26 types of Cancer in 1.44 million adults. JAMA Intern Med. 2016;176(6):816–25. 27183032 10.1001/jamainternmed.2016.1548 PMC5812009

[40] Ulf Ekelund WJ, Brown J, Steene-Johannessen MW, Fagerland N, Owen KE, Powell, et al. Do the associations of sedentary behaviour with cardiovascular disease mortality and cancer mortality differ by physical activity level? A systematic review and harmonised meta-analysis of data from 850 060 participants. Br J Sports Med. 2019;53(14):886. 29991570 10.1136/bjsports-2017-098963

[41] World Health Organization. Global action plan on physical activity 2018–2030: more active people for a healthier world [Internet]. World Health Organization. 2019. https://www.un.org/development/desa/dspd/wp-content/uploads/sites/22/2019/09/WHO_GAPPA_2018-2030.pdf

[42] Katzmarzyk PT, Church TS, Craig CL, Bouchard C. Sitting time and mortality from all causes, cardiovascular disease, and cancer. Med Sci Sports Exerc. 2009;41(5):998–1005. 19346988 10.1249/MSS.0b013e3181930355

[43] Gao W, Sanna M, Chen YH, Tsai MK, Wen CP. Occupational sitting time, Leisure Physical Activity, and all-cause and Cardiovascular Disease Mortality. JAMA Netw Open. 2024;7(1):e2350680–2350680. 38241049 10.1001/jamanetworkopen.2023.50680 PMC10799265

[44] Kazemi A, Soltani S, Aune D, Hosseini E, Mokhtari Z, Hassanzadeh Z, et al. Leisure-time and occupational physical activity and risk of cardiovascular disease incidence: a systematic-review and dose-response meta-analysis of prospective cohort studies. Int J Behav Nutr Phys Act. 2024;21(1):45. 38659024 10.1186/s12966-024-01593-8 PMC11044601

[45] Suutari-Jääskö A, Parkkila K, Perkiömäki J, Huikuri H, Kesäniemi YA, Ukkola OH. Leisure time and occupational physical activity, overall and cardiovascular mortality: a 24-year follow-up in the OPERA study. Ann Med. 2023;55(2):2245429. 37585501 10.1080/07853890.2023.2245429 PMC10435002

[46] Fluharty ME, Pinto Pereira SM, Benzeval M, Hamer M, Jefferis B, Griffiths LJ, et al. Educational differentials in key domains of physical activity by ethnicity, age and sex: a cross-sectional study of over 40 000 participants in the UK household longitudinal study (2013–2015). BMJ Open. 2020;10(1):e033318. 31964672 10.1136/bmjopen-2019-033318 PMC7045199

[47] Scholes S, Bann D. Education-related disparities in reported physical activity during leisure-time, active transportation, and work among US adults: repeated cross-sectional analysis from the National Health and Nutrition Examination Surveys, 2007 to 2016. BMC Public Health. 2018;18(1):926. 30055611 10.1186/s12889-018-5857-z PMC6064072

[48] Al-Mallah MH, Juraschek SP, Whelton S, Dardari ZA, Ehrman JK, Michos ED et al. Sex Differences in Cardiorespiratory Fitness and All-Cause Mortality: The Henry Ford ExercIse Testing (FIT) Project. Mayo Clin Proc. 2016;91(6):755–62. 10.1016/j.mayocp.2016.04.002 PMC5617114 27161032

[49] Ji H, Gulati M, Huang TY, Kwan AC, Ouyang D, Ebinger JE, et al. Sex differences in Association of Physical Activity with all-cause and Cardiovascular Mortality. J Am Coll Cardiol. 2024;83(8):783–93. 38383092 10.1016/j.jacc.2023.12.019 PMC10984219

[50] Mclaughlin M, Atkin AJ, Starr L, Hall A, Wolfenden L, Sutherland R, et al. Worldwide surveillance of self-reported sitting time: a scoping review. Int J Behav Nutr Phys Act. 2020;17(1):111. 32883294 10.1186/s12966-020-01008-4 PMC7469304

[51] Nidhi Gupta CS, Christiansen C, Hanisch H, Bay H, Burr A, Holtermann. Is questionnaire-based sitting time inaccurate and can it be improved? A cross-sectional investigation using accelerometer-based sitting time. BMJ Open. 2017;7(1):e013251. 28093433 10.1136/bmjopen-2016-013251 PMC5253534

[52] Lee PH, Macfarlane DJ, Lam TH, Stewart SM. Validity of the International Physical Activity Questionnaire Short Form (IPAQ-SF): a systematic review. Int J Behav Nutr Phys Act. 2011;8:115. 22018588 10.1186/1479-5868-8-115 PMC3214824

[53] Sember V, Meh K, Sorić M, Starc G, Rocha P, Jurak G. Validity and reliability of International Physical Activity questionnaires for adults across EU countries: systematic review and Meta Analysis. Int J Environ Res Public Health. 2020;17:19. 10.3390/ijerph17197161 PMC7579664 33007880

[54] Meh K, Jurak G, Sorić M, Rocha P, Sember V. Validity and reliability of IPAQ-SF and GPAQ for assessing sedentary behaviour in adults in the European Union: a systematic review and Meta-analysis. Int J Environ Res Public Health. 2021;18(9). 10.3390/ijerph18094602 PMC8123682 33926123

[55] Milton K, Clemes S, Bull F. Can a single question provide an accurate measure of physical activity? Br J Sports Med. 2013;47(1):44–8. 22522584 10.1136/bjsports-2011-090899

[56] Lee IM, Shiroma EJ. Using accelerometers to measure physical activity in large-scale epidemiological studies: issues and challenges. Br J Sports Med. 2014;48(3):197–201. 24297837 10.1136/bjsports-2013-093154 PMC3947179

[57] Burchartz A, Manz K, Anedda B, Niessner C, Oriwol D, Schmidt SC, et al. Measurement of physical activity and sedentary behavior by Accelerometry among a Nationwide Sample from the KiGGS and MoMo Study: study protocol. JMIR Res Protoc. 2020;9(7):e14370. 32459648 10.2196/14370 PMC7388053

[58] Young DR, Hivert MF, Alhassan S, Camhi SM, Ferguson JF, Katzmarzyk PT, et al. Sedentary Behavior and Cardiovascular Morbidity and Mortality: A Science Advisory from the American Heart Association. Circulation. 2016;134(13):e262–79. 27528691 10.1161/CIR.0000000000000440

